# Protective Effects of* Panax notoginseng* Saponins against High Glucose-Induced Oxidative Injury in Rat Retinal Capillary Endothelial Cells

**DOI:** 10.1155/2016/5326382

**Published:** 2016-02-25

**Authors:** Yue Fan, Yuan Qiao, Jianmei Huang, Minke Tang

**Affiliations:** School of Chinese Material, Beijing University of Chinese Medicine, Beijing 100102, China

## Abstract

Diabetic retinopathy, a leading cause of visual loss and blindness, is characterized by microvascular dysfunction. Hyperglycemia is considered the major pathogenic factor for diabetic retinopathy and is associated with increased oxidative stress in the retina. In this study, we investigated the potential protective effects of* Panax notoginseng* Saponins (PNS) in retinal capillary endothelial cells (RCECs) exposed to high glucose conditions. We found a pronounced increase in cell viability in rat RCECs incubated with both PNS and high glucose (30 mM) for 48 h or 72 h. The increased viability was accompanied by reduced intracellular hydrogen peroxide (H_2_O_2_) and superoxide (O_2_^−^), decreased mitochondrial reactive oxygen species (ROS), and lowered malondialdehyde (MDA) levels. PNS also increased the activities of total superoxide dismutase (SOD), MnSOD, catalase (CAT), and glutathione peroxidase (GSH-PX). The glutathione (GSH) content also increased after PNS treatment. Furthermore, PNS reduced NADPH oxidase 4 (Nox4) expression. These results indicate that PNS exerts a protective effect against high glucose-induced injury in RCECs, which may be partially attributed to its antioxidative function.

## 1. Introduction

Diabetic retinopathy is a common complication of diabetes mellitus and a leading cause of acquired blindness in adults in developed countries. Diabetic retinopathy is characterized by progressive alterations in the retinal microvasculature. Retinal microvascular dysfunction is clinically characterized by the loss of endothelial cells and pericytes, capillary occlusion, and blood-retinal barrier (BRB) breakdown during the early stages [1, 2]. Hyperglycemia is considered the main contributing factor for developing diabetic retinopathy, triggering a cascade of pathological metabolic and biochemical changes. Several reports demonstrate that hyperglycemia increased damage in microvascular cells in the retina [3, 4].

The retina has high polyunsaturated fatty acid content and the highest relative oxygen uptake and glucose oxidation when compared to many tissues; these phenomena render the retina more susceptible to oxidative stress [5]. Indeed, a large body of evidence supports the idea that an oxidative stress increase in the retinal microvasculature is a key factor for developing diabetic retinopathy [3]. Endothelial cells, similar to other nonphagocytic cells, generate reactive oxygen species (ROS), including superoxide (O_2_^−^) and hydrogen peroxide (H_2_O_2_). Although low concentrations of ROS might serve as intracellular signaling molecules to induce repair mechanisms against tissue injury, large amounts of ROS are considered toxic products that can cause cell death [6]. In diabetes, the activities of antioxidant defense enzymes are responsible for scavenging free radicals and maintaining redox homeostasis. Superoxide dismutase (SOD) is considered a first-line defense against ROS. This enzyme is present in nearly all cells and converts O_2_^−^ into H_2_O_2_. As H_2_O_2_ can react with ROS, it is further degraded by one of two antioxidant enzymes, glutathione peroxidase (GSH-PX) or catalase (CAT) [7, 8]. Furthermore, the cell is equipped with glutathione (GSH), an intracellular antioxidant that is probably the most important defense in the cell. It can act as an ROS scavenger and modulate the intracellular redox state [9].

*Panax notoinseng*, known as Sanqi in Chinese, is mainly cultivated in the Yunnan and the Guangxi provinces of China. The medicinal properties of the* Panax notoginseng* root include promoting blood clotting, relieving swelling, and alleviating pain [10].* Panax notoginseng* Saponins (PNS) is the main active chemical ingredient of* Panax notoginseng*, which is mainly composed of ginsenoside Rg1, ginsenoside Rb1, notoginsenoside R1, and so forth, and belongs to the Araliaceae family. Numerous studies reported that PNS had a protective effect on oxidative stress-induced damage and apoptosis in cultured rabbit bone marrow stromal cells (BMSCs) [11], primary astrocytes, and a neuroblastoma cell line, SH-SY5Y [12]. We postulated that PNS executes a protective function against high glucose-induced injury in RCECs, which may be partially attributed to its antioxidative properties. Therefore, we evaluated the effect of PNS on ROS; MDA content; the activities of total SOD, MnSOD, CAT, and GSH-PX; GSH content; and expression of Nox4 in rat RCECs treated with high glucose. The present findings provide evidence for a functional role of PNS in RCECs in the prevention of diabetic retinopathy.

## 2. Materials and Methods

### 2.1. Animals

Male Sprague-Dawley (SD) rats weighing 180–200 g were obtained from the Vital River Laboratory Animal Technology Co., Ltd., in Beijing, China. The certificate number was SCXK (Jing) 2012-0001. The laboratory animal care guidelines, approved by the Animal Ethics Committee at the Beijing University of Chinese Medicine, were strictly followed. All efforts were made to minimize animal suffering and to reduce the number of animals used for the experiments.

### 2.2. Cell Culture and Identification

Capillary endothelial cells derived from rat retinas and confirmed to be positive for vWf and CD31 proteins by immunofluorescence microscopy were used in this study. To establish the primary cell culture of RCECs, retinas isolated from 20 rat eyes were minced into small pieces, washed in Phosphate Buffered Saline (PBS) containing 5% penicillin/streptomycin, with the retinal fragments retained on a 100 *μ*m cell strainer. Following harvesting, the fragments from sieve were incubated in 3 mL of 0.1% type II collagenase (Sigma Aldrich St. Louis, MI, USA) at 37°C for 30 min with agitation. The retinal fragment suspension was filtered through a 70 *μ*m cell strainer. After centrifugation at 174 ×g for 5 min, the pellets containing microvessel fragments were suspended in endothelial cell medium (ECM, Sciencell, San Diego, CA, USA). The above suspension was transferred to T-25 cm^2^ flask precoated with 1% gelatin (Gibco, Life Technologies Inc., Grand Island, NY, USA) and cultured at 37°C in a 5% CO_2_ humidified incubator. After 48 h, half of the medium was renewed and thereafter the medium was changed every 2-3 days. The confluent cultures were passaged by detaching the cells using 0.1% trypsin (Sigma Aldrich, St. Louis, MI, USA) in PBS for 2-3 min and plating at a split ratio of 1 : 2. The cells used in this study were passaged 3–5 times.

Endothelial cells were stained with primary antibodies recognizing vWf and CD31. Briefly, the cells were fixed in 4% paraformaldehyde (PFA) for 30 min at room temperature. After fixation, the cells were washed with PBS three times and blocked with normal goat serum (NGS) for 30 min. The cells were then incubated with primary antibodies (rabbit anti-vWf, 1 : 100; rabbit anti-CD31, 1 : 100, Santa Cruz, Dallas, Texas, USA) at 4°C overnight. The cells were then washed with PBS and probed with FTIC-labeled anti-rabbit secondary antibody (1 : 100, Zhong Shan Jin Qiao, Beijing, China). 4′,6-Diamidino-2-phenylindole (DAPI, Cell Signaling Technology, Danvers, MA, USA) was used to stain nuclei in the final step. The images were captured using a fluorescence microscope.

### 2.3. MTT Assay

The rat RCECs were seeded in gelatin-coated 96-well plates, allowed to attach overnight, and then incubated with either 5.5 mM of glucose or 30 mM of glucose with or without varying concentrations of PNS (20, 50, 100, and 200 *μ*g/mL) for 24 h, 48 h, and 72 h. Four hours before the culture was terminated, MTT (5 mg/mL) was added to each well. After 4 h of incubation with MTT and medium at 37°C, the supernatants were removed and 150 *μ*L of dimethylsulfoxide [(DMSO), Sigma Aldrich, St. Louis, MI, USA] was added to each well to dissolve the blue formazan. The 540 nm absorbance of each well was read on an enzyme-linked immunosorbent assay reader (Thermo Labsystems, Finland). All of the experiments were repeated three times.

### 2.4. Trypan Blue Staining Assay

Rat RCECs were seeded in gelatin-coated 24-well plates, allowed to attach overnight, and then exposed to experimental conditions. After treatment, the supernatant was removed, 0.1% trypsin was added to each well to suspend the cells, and then 0.4% trypan blue solution (Sigma Aldrich, St. Louis, MI, USA) was added to each well to stain the cells. The cell numbers were observed by an inverted microscope (LEICA, Germany).

### 2.5. Fluorescent Probe Assay

The fluorescent probes DCFH-DA (Sigma Aldrich, St. Louis, MI, USA), DHE (Sigma Aldrich, St. Louis, MI, USA), and MitoTracker Red CM-H_2_XRos (Invitrogen, Life Technologies Inc., Grand Island, NY, USA) were used to measure the production of intracellular H_2_O_2_ and O_2_^−^ and mitochondrial ROS. Briefly, confluent rat RCECs plated in 35 mm^2^ confocal laser special disks were exposed to varying experimental conditions. The reactions were stopped by removing the medium, washing with PBS, staining with DCFH-DA (at a final concentration of 10 *μ*M for 30 minutes), DHE (at a final concentration of 5 *μ*M for 30 minutes), and MitoTracker Red CM-H_2_XRos (at a final concentration of 2.5 *μ*M for 20 minutes) at 37°C, washing with PBS, and then observing under an ECLIPSE Ti laser confocal microscope (Nikon, Japan). Confocal images were processed with ImageJ software.

### 2.6. Biochemical Assay

Rat RCECs were seeded in gelatin-coated T-25 cm^2^ flasks and allowed to attach for 24 h before treatment. After 72 h of treatment, the cells were harvested in lysis buffer [50 mM Tris (pH 7.5), 150 mM NaCl, 1% NP40, 0.5% sodium deoxycholate, 1 mM EDTA, and 0.1% SDS], and the protein concentrations were determined by a BCA protein assay. Aliquots were stored at −80°C until use in assays to detect MDA, SOD, Mn-SOD, CAT, GSH-PX, GSH, and GSSG. MDA SOD, Mn-SOD, CAT, and GSH-PX were detected using the respective kits (Nanjing, Jiancheng Bioengineering Institute, Nanjing, China) and according to the manufacturer's instructions. The MDA content was measured through a reaction with thiobarbituric acid that forms a stable chromophoric product, visible at a wavelength of 532 nm. The MDA levels were expressed as nanomoles per milligram of protein. The SOD activity was measured according to its ability to inhibit the production of a water-soluble formazan dye. The CAT activity was determined by the conversion rate of hydrogen peroxide into H_2_O and O_2_. The GSH-PX activity was determined by measuring the rate of oxidation of GSH to GSSG, which is monitored by the dismutation of cumene hydroperoxide that is then catalyzed by GSH-PX. The total SOD, MnSOD, CAT, and GSH-PX activities were expressed as units per milligram of protein. GSH and GSSG contents were determined using commercially available kits (Beyotime Biotech, Shanghai, China). All of the procedures complied with the manufacturer's instructions. GSH and GSSG levels were expressed in nanomoles per milligram of protein.

### 2.7. Immunofluorescence

Rat RCECs were seeded in gelatin-coated 24-well plates, allowed to attach overnight, and then exposed to various experimental conditions. After treatment, the rat RCECs were fixed in 4% PFA for 30 min at room temperature. After fixation, the cells were washed with PBS and permeabilized with 0.1% Triton X-100 for 10 min. Nonspecific binding sites were blocked using a 30 min incubation NGS. The cells were incubated with primary antibody (rabbit polyclonal anti-Nox4, 1 : 50, Santa Cruz, Dallas, Texas, USA) at 4°C overnight. Cells were then washed with PBS and incubated with FITC-labeled secondary antibody (goat anti-rabbit IgG/FITC, 1 : 100, Zhong Shan Jin Qiao, Beijing, China) for 1 h at 37°C. DAPI was used to stain nuclei in the final incubation step, and then the cells were observed under an ECLIPSE Ti laser confocal microscope (Nikon, Japan). Confocal images were processed with ImageJ software.

### 2.8. Western Blot Analysis

Rat RCECs were seeded in gelatin-coated T-25 cm^2^ flasks, allowed to attach overnight, and then exposed to varying experimental conditions. Cells were harvested in lysis buffer [50 mM Tris (pH 7.5), 150 mM NaCl, 1% NP40, 0.5% sodium deoxycholate, 1 mM EDTA and 0.1% SDS], and the protein concentrations were determined by a BCA protein assay. The protein (8 *μ*g) was electrophoresed on a 10% SDS-polyacrylamide gel, transferred to a polyvinylidene difluoride (PVDF) membrane, and incubated with primary antibody (rabbit polyclonal anti-Nox4, 1 : 500, Santa Cruz, Dallas, Texas, USA) at 4°C overnight. After washing, the membranes were incubated with horseradish peroxidase-conjugated secondary antibody for 40 min. The immunoreactive bands were visualized using an enhanced chemiluminescence kit. The relative densities of the bands were determined by image analysis with Image-Pro Plus software.

### 2.9. Statistical Analysis

All of the data are presented as the mean value ± standard deviation (SD). One-way ANOVA, followed by a Student-Newman-Keuls test was used to analyze all of the data. A value of *P* < 0.05 and *P* < 0.01 was considered significant. The statistical analyses were performed using SPSS 17.0 software (SPSS, Chicago, USA).

## 3. Results 

### 3.1. Validation of Rat RCECs

**Figure 1:**
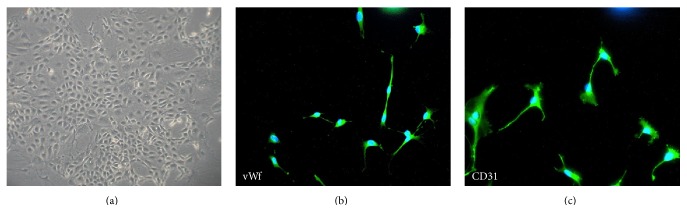
Morphology and immunofluorescence in RCECs. RCECs from rat retinal microvascular fragments were cultured in endothelial cell medium. When the RCECs were cultured for 10 days, the characteristic cobble-stone morphology of RCECs was observed (a). The RCECs were positive for both vWf (b, green fluorescent) and CD31 (c, green fluorescent), as determined by an immunofluorescent assay. All nuclei were stained with DAPI (blue fluorescent).

In this study, the cells showed a cobble-stone morphology and a contact-inhibited monolayer (Figure 1(a)) when observed under an inverted microscope. The cells expressed von Willebrand factor (vWf) (Figure 1(b)) and platelet endothelial cell adhesion molecule-1 (PECAM-1/CD31) (Figure 1(c)), which are well-known endothelial cell-specific markers.

### 3.2. PNS Increases Cell Viability in Rat RCECs Exposed to High Glucose

**Figure 2:**
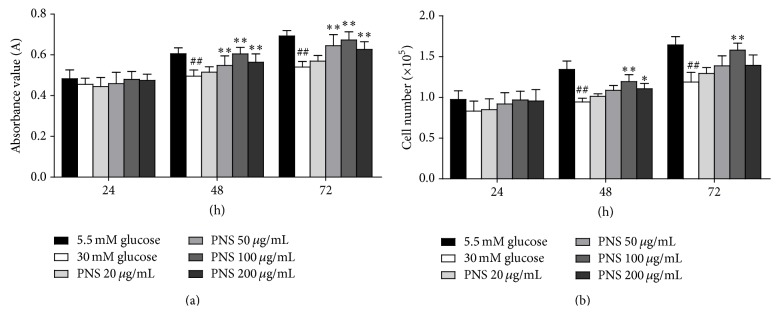
PNS increased cell viability in rat RCECs exposed to high glucose. Seeded in 24-well or 96-well plates, the rat RCECs were incubated with varying concentrations of PNS (20, 50, 100, and 200 *μ*g/mL) in 30 mM glucose. After culturing for 24 h, 48 h, or 72 h, MTT and trypan blue staining assay were used to examine cell viability. The MTT assay showed that 50, 100, and 200 *μ*g/mL of PNS increased cell viability after 48 and 72 h (a). The trypan blue assay showed that 100 and 200 *μ*g/mL of PNS increased cell viability after 48 h and that 100 *μ*g/mL of PNS increased cell viability after 72 h (b). The experiment was repeated three times. Data are expressed as the mean ± SD (*n* = 5). ^##^ *P* < 0.01 versus 5.5 mM glucose; ^*∗∗*^ *P* < 0.01 and ^*∗*^ *P* < 0.05 versus 30 mM glucose.

In this study, we observed that stimulation with 30 mM glucose for 48 h and 72 h significantly decreased cell viability in comparison with 5.5 mM glucose-treated cells (*P* < 0.01). In the MTT assay, 50 *μ*g/mL, 100 *μ*g/mL, and 200 *μ*g/mL of PNS significantly increased cell viability (*P* < 0.01) after 48 h or 72 h of treatment (Figure 2(a)). In the trypan blue assay, we found that 100 *μ*g/mL and 200 *μ*g/mL of PNS increased cell viability after 48 h of treatment (*P* < 0.05), and 100 *μ*g/mL of PNS increased cell viability after 72 h of treatment (*P* < 0.01) (Figure 2(b)). These data suggest that PNS could increase cell viability in high glucose-treated RCECs.

### 3.3. PNS Inhibits the High Glucose-Induced Increase in ROS and MDA in Rat RCECs

**Figure 3:**
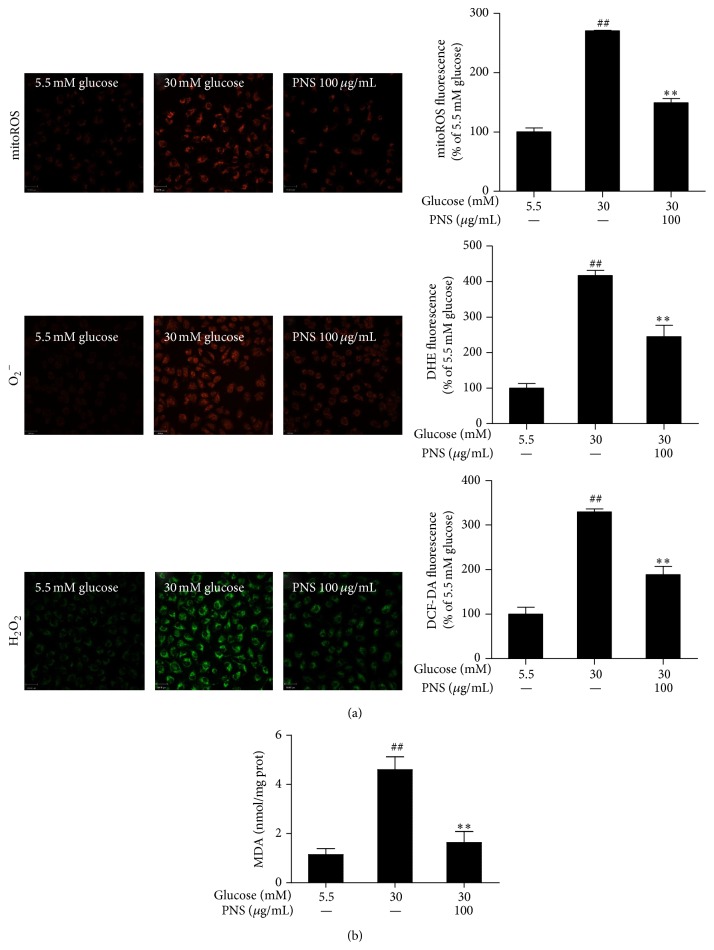
PNS inhibited the ROS and MDA induced by high glucose in rat RCECs. Seeded in plates or flasks, rat RCECs were incubated with PNS (100 *μ*g/mL) in 30 mM glucose for 72 h. Fluorogenic probes (DCFH-DA, DHE, and MitoTracker Red CM-H_2_XRos) showed that, after 72 h of PNS treatment, the intracellular H_2_O_2_ and O_2_^−^ and mitochondrial ROS were decreased (a). A lipid peroxidation assay showed that PNS could decrease the MDA content (b). Data are expressed as the mean ± SD (*n* = 4). ^##^ *P* < 0.01 versus 5.5 mM glucose; ^*∗∗*^ *P* < 0.01 versus 30 mM glucose. Scale bar: 33 *μ*m.

As shown in Figure 3, 30 mM glucose significantly enhanced intracellular H_2_O_2_ and O_2_^−^ and mitochondrial ROS (*P* < 0.01), observed by staining with 3 different fluorogenic probes: dichloro-dihydro-fluorescein diacetate (DCFH-DA), dihydroethidium (DHE) and MitoTracker Red CM-H_2_XRos. PNS at 100 *μ*g/mL reduced the 30 mM glucose-induced increase in intracellular H_2_O_2_ an, O_2_^−^ and mitochondrial ROS (*P* < 0.01). The malondialdehyde (MDA) content was significantly increased after 72 h of 30 mM glucose treatment (*P* < 0.01) compared with cells treated with 5.5 mM glucose. Treatment with PNS (100 *μ*g/mL) inhibited the elevated MDA observed in RCECs treated with 30 mM glucose (*P* < 0.01). These data suggest that PNS significantly reduced the levels of ROS and MDA, showing an effective attenuation of high glucose-induced oxidative injury.

### 3.4. PNS Increases SOD and CAT Activity and Regulates Glutathione Redox in Rat RCECs Exposed to High Glucose

**Figure 4:**
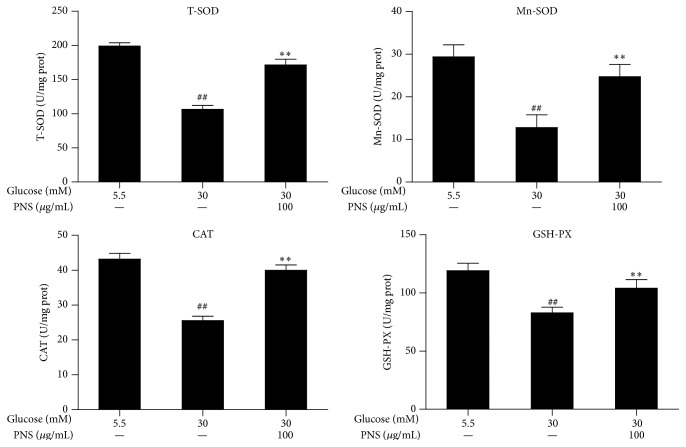
PNS increased the activities of total SOD, Mn-SOD, CAT, and GSH-PX in rat RCECs exposed to high glucose. Rat RCECs were incubated with concentration of PNS (100 *μ*g/mL) in 30 mM glucose for 72 h; cells were collected and subjected for total SOD, MnSOD, CAT, and GSH-PX determination. The results showed that PNS increased the activities of total SOD, MnSOD, CAT, and GSH-PX in rat RCECs incubated with 30 mM glucose. Data are expressed as the mean ± SD (*n* = 4). ^##^ *P* < 0.01 versus 5.5 mM glucose; ^*∗∗*^ *P* < 0.01 versus 30 mM glucose.

**Figure 5:**
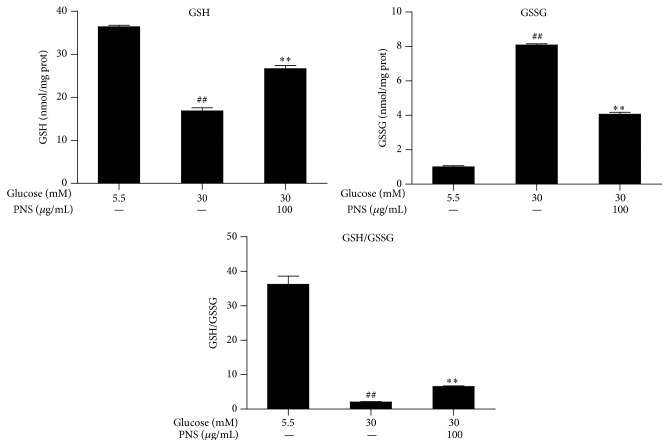
PNS increased GSH and reduced GSSG contents in rat RCECs exposed to high glucose. Rat RCECs were incubated with PNS (100 *μ*g/mL) in 30 mM of glucose for 72 h; then cells were collected and GSH and GSSG were quantified. The results showed that PNS increased GSH content and reduced GSSG content after incubation with 30 mM of glucose. Data are expressed as the mean ± SD (*n* = 4). ^##^ *P* < 0.01 versus 5.5 mM glucose; ^*∗∗*^ *P* < 0.01 versus 30 mM glucose.

The total SOD, Mn-SOD, CAT, and GSH-PX activity in the RCECs significantly decreased when exposed to 30 mM glucose compared with 5.5 mM glucose (*P* < 0.01). Treatment with PNS (100 *μ*g/mL) increased the total SOD, Mn-SOD, CAT, and GSH-PX activity (*P* < 0.01), as shown in Figure 4. When exposed to 30 mM glucose, the GSH level in the RCECs markedly decreased while the GSSG level increased, subsequently enhancing the GSH/GSSG ratio (*P* < 0.01). Treatment with PNS (100 *μ*g/mL) increased GSH levels, decreased GSSH levels, and enhanced the GSH/GSSG ratio (*P* < 0.01) (Figure 5). These data suggest that PNS had significant antioxidative effects against high glucose-induced oxidative stress in rat RCECs.

### 3.5. PNS Inhibited the NADPH Oxidase 4 (Nox4) Expressions in Rat RCECs Exposed to High Glucose

**Figure 6:**
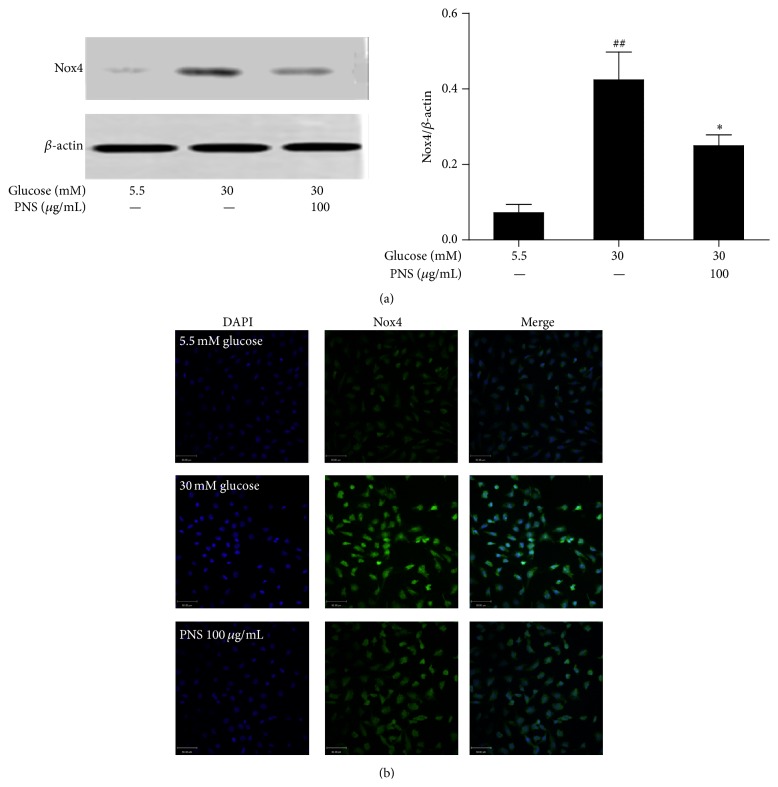
PNS inhibited NADPH oxidase 4 (Nox4) expression in rat RCECs exposed to high glucose. Western blot (a) and immunofluorescence (b) analyses were performed to detect Nox4 by PNS in rat RCECs exposed to 30 mM of glucose for 72 h. We found that PNS could reduce Nox4 expression. Data are expressed as the mean ± SD (*n* = 4). ^##^ *P* < 0.01 versus 5.5 mM glucose; ^*∗*^ *P* < 0.05 versus 30 mM glucose. Scale bar: 50 *μ*m.

As shown in Figure 6, western blot and immunofluorescence showed increased expression of Nox4 protein in the rat RCECs treated with 30 mM glucose (*P* < 0.01). Treatment with PNS (100 *μ*g/mL) markedly inhibited the high glucose-induced increase of Nox4 expression (*P* < 0.05).

## 4. Discussion 

In this study, we found that PNS treatment attenuates high glucose-induced injury in rat RCECs and is accompanied by reduced intracellular ROS and lowered MDA contents. PNS increased the intracellular SOD, mitochondrial SOD, CAT, and GSH-PX activities. The GSH content also increased after PNS treatment. These results indicate that PNS may exert its protective effect through the promotion of antioxidation.

Diabetic retinopathy is characterized by progressive alterations in the retinal microvasculature, and hyperglycemia is considered the prime triggering factor for increased BRB permeability [13]. Studies have found that rat RCECs [14], human RCECs [15], or bovine RCECs [16] display increased apoptosis when cells are exposed to high glucose. Similar results were seen in the TR-iBRB2 rat RCEC cell line [17]. Here, we employed a cell-based experimental model in which cultured RCECs were exposed to 30 mM glucose and confirmed the detrimental effect of hyperglycemia on the rat RCEC functionality. At same time, the coincubation with PNS improved RCEC viability, indicating that PNS protected the RCECs against high glucose-induced injury.

A large body of evidence has demonstrated an increase in ROS in different tissue and cell types during diabetes or after exposure to high glucose [18, 19]. These data are the basis for the claim that high glucose contributes to the vascular alterations observed in diabetic retinopathy. Some studies have shown that mitochondrial ROS levels are increased in diabetic rat retinas and in retinal cells incubated in high glucose and that downregulation of mitochondrial ROS can inhibit glucose-induced apoptosis in both endothelial cells and pericytes [3, 4]. In this study, we found that, after culturing RCECs 30 mM glucose medium for 72 h, the mitochondria ROS was significantly increased and intracellular H_2_O_2_ and O_2_ were concomitantly increased. High glucose increased MDA, which suggests that hyperglycemia can increase oxidative damage in RCECs, an observation that agrees with previous studies. PNS reduced intracellular H_2_O_2_ and O_2_^−^ and mitochondrial ROS, as indicated by the decreased fluorescence intensity of DCF-DA, DHE, and MitoTracker Red CM-H_2_XRos. Meanwhile, PNS markedly decreased the MDA levels. These data suggest that PNS may exert antioxidant effects in the intracellular compartment. It should be acknowledged that intracellular ROS may come from nonmitochondrial sources, although the mitochondrion is main source. Previous studies have confirmed that Nox4, a homolog of gp91phox/Nox2, was abundantly expressed in endothelial cells. Overexpression of Nox4 can cause an imbalance between the production of free radicals and the antioxidant defense system [18, 20]. Our study showed a significant increase in Nox4 expression after high glucose exposure, indicating elevated ROS production. PNS significantly reduced the Nox4 expression levels. These data suggest that PNS may reduce the glucose-induced ROS via decreasing the high glucose-induced Nox4 expression.

Oxidative stress causes cell damage when the antioxidant enzymes and antioxidative substrates are exhausted [21]. During oxidative stress, the glutathione redox system plays an important role in endothelial cells. This process has been reported in many other cell types and proposed as a mechanism of cellular self-defense [22]. Thus, in order to investigate the possible mechanism by which PNS protects RCECs from high glucose-induced oxidative injury, the glutathione and glutathione related enzymes were examined. The results indicated that PNS treatment caused a significant enhancement of the GSH content and GSH-PX activity. Undoubtedly, these contribute to the restored cell viability and the antioxidative capacity. In addition to the glutathione system, CAT is also an important antioxidant pathway in the removal of H_2_O_2_ [21]. The data show that PNS could decrease CAT activity. SOD, an endogenous antioxidant, catalyzes the breakdown of O_2_^−^ into H_2_O_2_ scavenging O_2_^−^ [22]. Because intramitochondrial O_2_^−^ does not readily cross mitochondrial membranes, MnSOD, a superoxide scavenging enzyme in mitochondria, converts intramitochondrial O_2_^−^ into H_2_O_2_ that can diffuse out of mitochondria [21]. In our study, PNS treated RCECs inhibited the SOD and MnSOD levels induced by 30 mM glucose. Thereby, it was reasonably presumed that reducing the ROS overload, coupled with improving antioxidant enzymes activities and glutathione redox system, may be the main protection mechanism of PNS in RCECs.

In conclusion, our findings indicated that PNS is endowed with a significant protective function against high glucose-induced oxidative injury in RCECs. The protective effect of PNS may be due to its ability to reduce oxidative stress. Therefore, it is plausible to conduct further investigation aimed at the clinical application of PNS in the treatment of diabetic retinopathy.
